# Author Correction: Genomic risk score offers predictive performance comparable to clinical risk factors for ischaemic stroke

**DOI:** 10.1038/s41467-020-14717-y

**Published:** 2020-02-20

**Authors:** Gad Abraham, Rainer Malik, Ekaterina Yonova-Doing, Agus Salim, Tingting Wang, John Danesh, Adam S. Butterworth, Joanna M. M. Howson, Michael Inouye, Martin Dichgans

**Affiliations:** 1 Cambridge Baker Systems Genomics Initiative, Baker Heart and Diabetes Institute, Melbourne, VIC Australia; 2 Cambridge Baker Systems Genomics Initiative, Department of Public Health and Primary Care, University of Cambridge, Cambridge, UK; 3 Department of Clinical Pathology, University of Melbourne, Parkville, VIC Australia; 4 Institute for Stroke and Dementia Research, University Hospital, Ludwig-Maximilians-Universität LMU, Munich, Germany; 5 British Heart Foundation Cardiovascular Epidemiology Unit, Department of Public Health and Primary Care, University of Cambridge, Cambridge, UK; 6 Baker Heart and Diabetes Institute, Melbourne, VIC Australia; 7 Department of Mathematics and Statistics, La Trobe University, Melbourne, VIC Australia; 8 British Heart Foundation Centre of Research Excellence, University of Cambridge, Cambridge, UK; 9 National Institute for Health Research Blood and Transplant Research Unit in Donor Health and Genomics, University of Cambridge, Cambridge, UK; 10 National Institute for Health Research Cambridge Biomedical Research Centre, University of Cambridge and Cambridge University Hospitals, Cambridge, UK; 11 Health Data Research UK Cambridge, Wellcome Genome Campus and University of Cambridge, Cambridge, UK; 12 Department of Human Genetics, Wellcome Sanger Institute, Hinxton, UK; 13 The Alan Turing Institute, London, UK; 14 German Center for Neurodegenerative Diseases (DZNE), Munich, Germany; 15 Munich Cluster for Systems Neurology (SyNergy), Munich, Germany

**Keywords:** Genome-wide association studies, Personalized medicine, Stroke, Risk factors

Correction to: *Nature Communications* 10.1038/s41467-019-13848-1, published online 20 December 2019.

In the original version of this article, the affiliations of author Joanna Howson were incorrectly given as “British Heart Foundation Cardiovascular Epidemiology Unit, Department of Public Health and Primary Care, University of Cambridge, Cambridge, UK and Health Data Research UK Cambridge, Wellcome Genome Campus and University of Cambridge, Cambridge, UK”. The correct affiliations are “British Heart Foundation Cardiovascular Epidemiology Unit, Department of Public Health and Primary Care, University of Cambridge, Cambridge, UK and National Institute for Health Research Cambridge Biomedical Research Centre, University of Cambridge and Cambridge University Hospitals, Cambridge, UK”.

In addition, the original version of this Article omitted the following from the Competing Interests statement: Dr Howson became a full time employee of Novo Nordisk while the manuscript was under review at Nature Communications.

These have been corrected in both the PDF and HTML versions of this article.

