# Supplementary material for: Genomic risk score offers predictive performance comparable to clinical risk factors for ischaemic stroke
Source: Nat Commun. 2019 Dec 20;10:5819. doi: 10.1038/s41467-019-13848-1 (PMC6925280; doi:10.1038/s41467-019-13848-1)
Supplement: Supplementary file 1 — Supplementary Information [file 41467_2019_13848_MOESM1_ESM.pdf]

Supplementary Information

Genomic risk score offers predictive performance comparable to clinical risk factors for ischaemic stroke

Abraham et al.

Supplementary Figures

Supplementary Figure 1: Associations of individual GRSs with ischaemic stroke in the UKB derivation set.

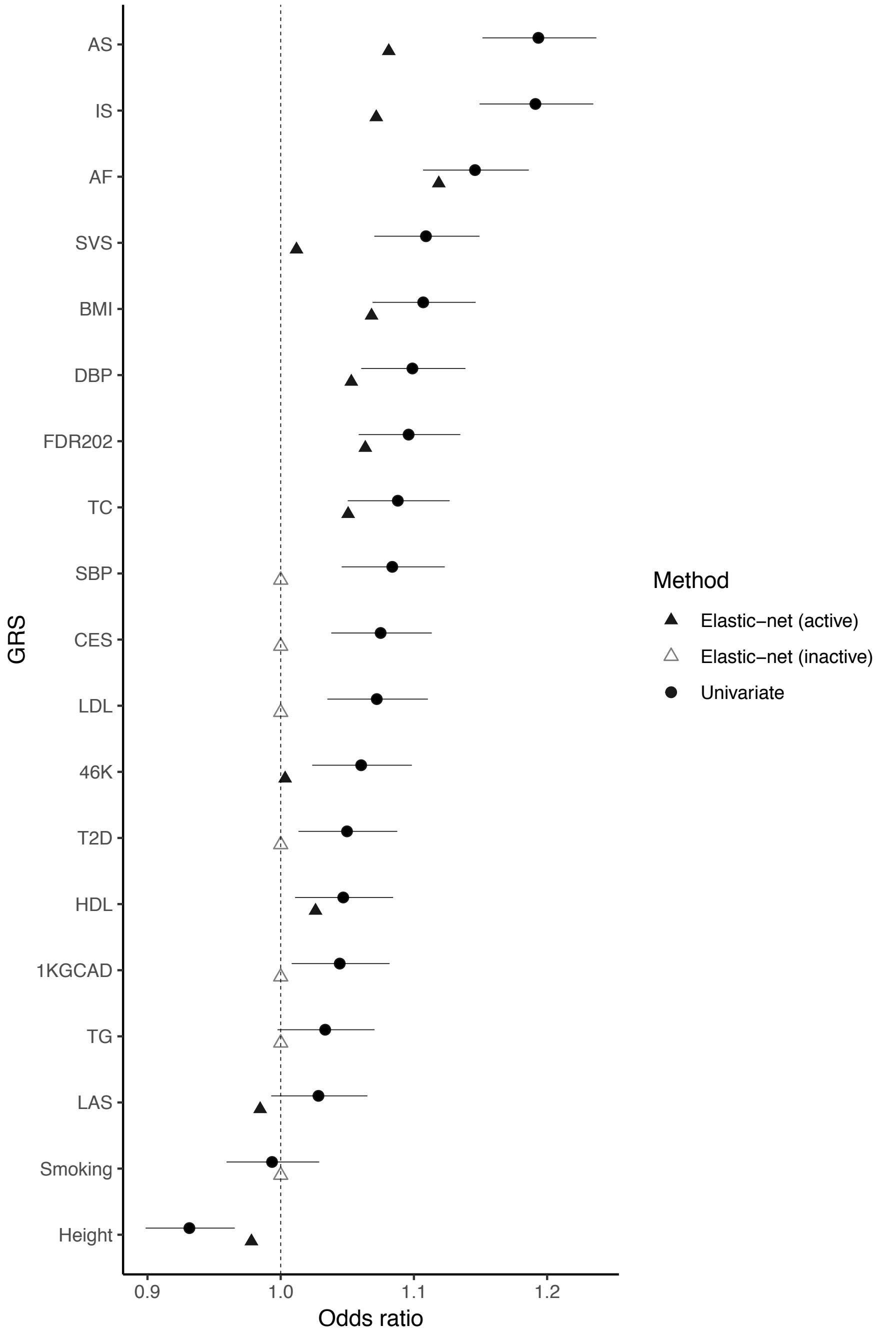

Shown are the odds ratios per standard deviation of each GRS estimated in either (i) logistic regression of each GRS, adjusting for chip (UKB/BiLEVE), 10 PCs, and sex (shown as filled circles); or in (ii) elastic-net logistic regression (shown as filled or empty triangles). The elastic-net estimates are from the best model selected via cross-validation, adjusting for all other GRSs, chip, sex, and 10 genetic PCs. For elastic-net, confidence intervals are not available; 'inactive' indicates that the elastic-net estimated odds ratio was negligible (between 0.999 and 1.001, shown as empty triangles). Scores are ordered by their univariate associations with ischaemic stroke. Error bars represent 95% confidence intervals.

Supplementary Figure 2: Comparison of the metaGRS approach with smtPred.

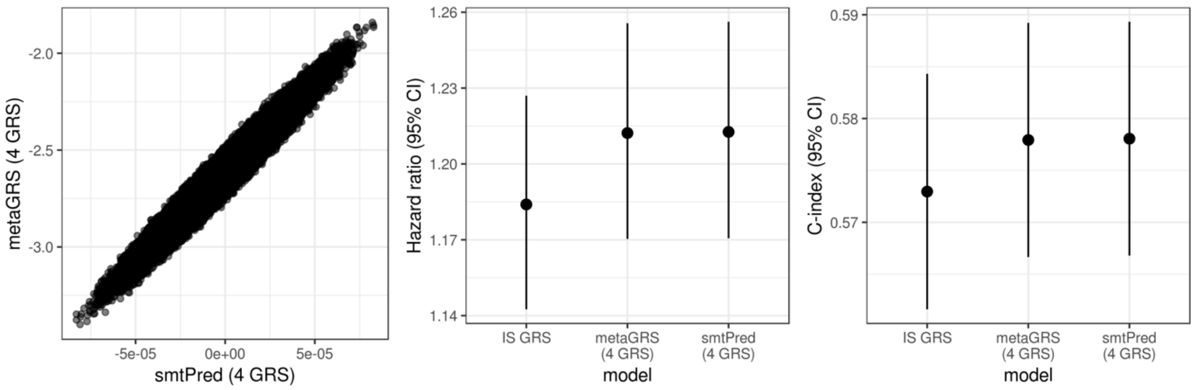

The metaGRS here is based on four component GRSs (Any Stroke [AS], Ischaemic Stroke [IS], Body Mass Index [BMI], and Systolic Blood Pressure [SBP]), and the smtPred model is based on the same four GRSs. The metaGRS was tuned in 10-fold cross-validation on the n=11,995 tuning set. Results are in the UK Biobank validation set (n=395,393). Error bars represent 95% confidence intervals.

Supplementary Figure 3: Comparison of the metaGRS with a published 90-SNP risk score for stroke[1] and the ischaemic stroke GRS derived earlier, predicting both prevalent and incident any stroke (AS) and ischaemic stroke (IS), in the UK Biobank validation set.

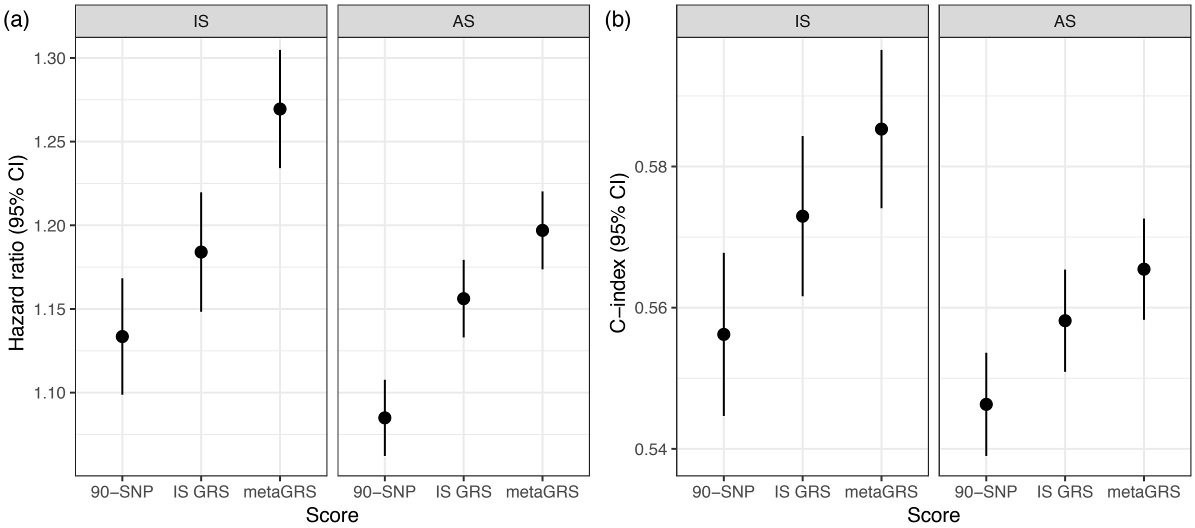

Scores were standardised to zero-mean, unit standard deviation. Results are from Cox regression on the UKB validation set (n=395,393), sex-stratified, adjusted for chip and 10 PCs. Error bars represent 95% confidence intervals.

Supplementary Figure 4: Cumulative incidence of ischaemic stroke stratified by the metaGRS (prevalent and incident stroke) in the UK Biobank validation set.

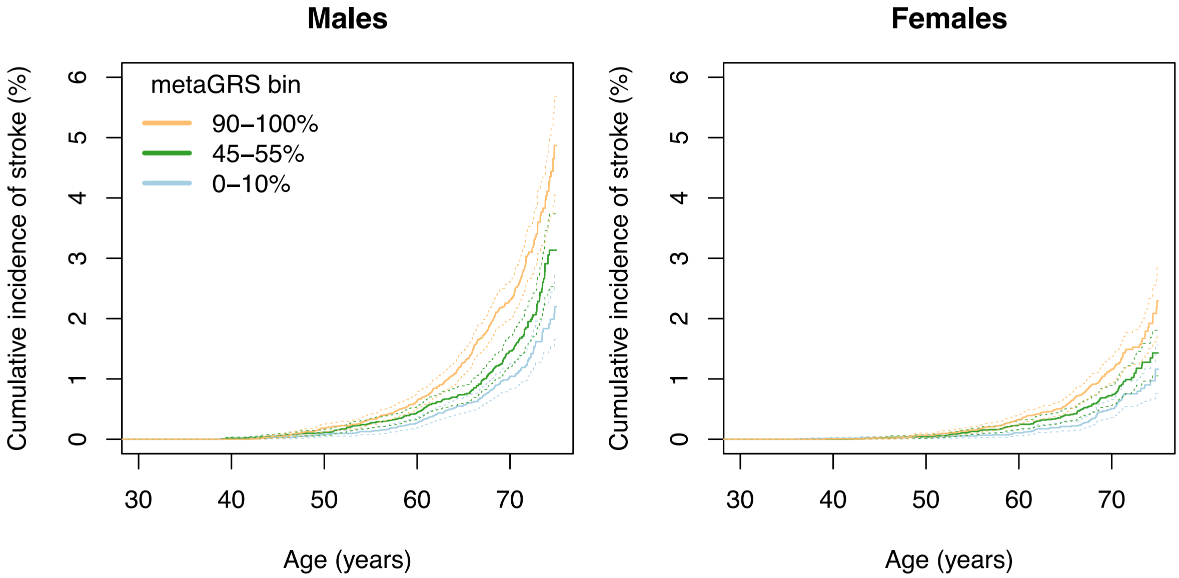

Results are from age-as-time-scale Kaplan-Meier analysis. Error bars represent 95% confidence intervals.

Supplementary Figure 5: Hazard ratios for different quantiles of the metaGRS (relative to the 45–55 centiles of the metaGRS distribution) for all recorded ischaemic stroke (prevalent and incident) in the UK Biobank validation set.

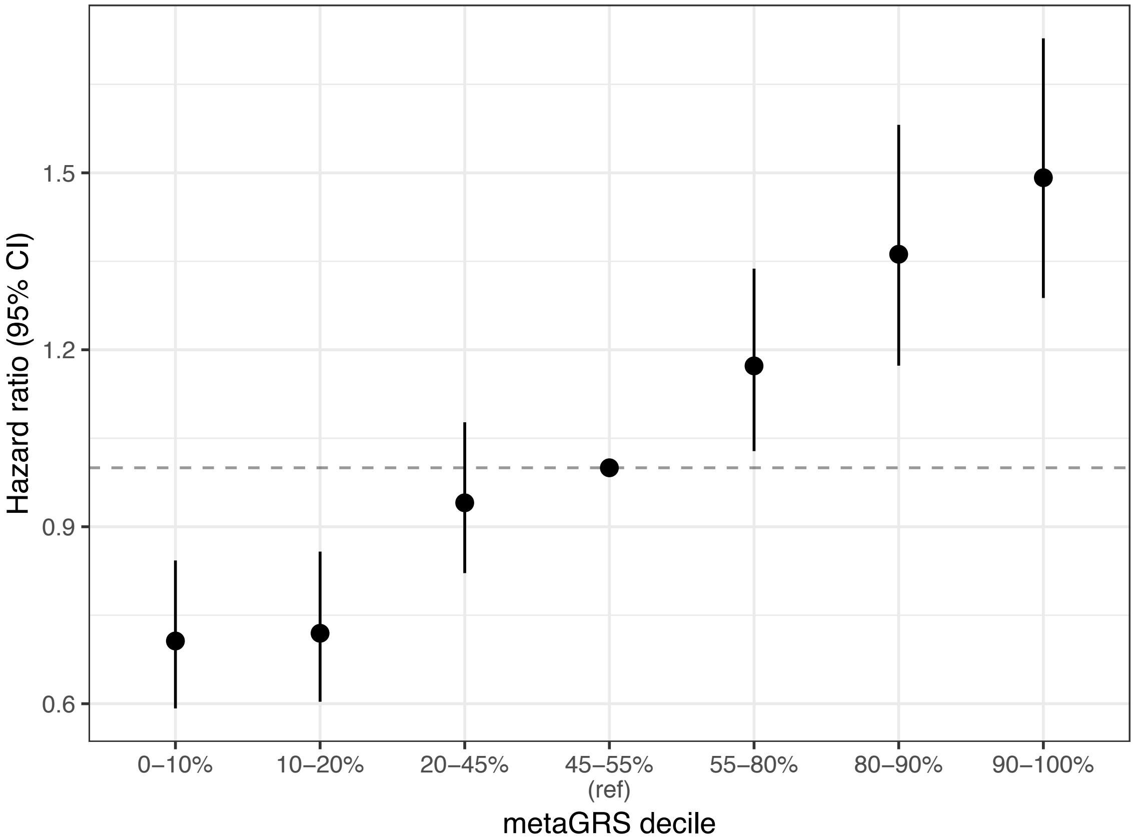

Based on age-as-time-scale Cox regression stratified by sex and adjusted for chip and 10 genetic PCs. Error bars represent 95% confidence intervals.

Supplementary Figure 6: Comparison of the 19-component metaGRS with smaller metaGRSs.

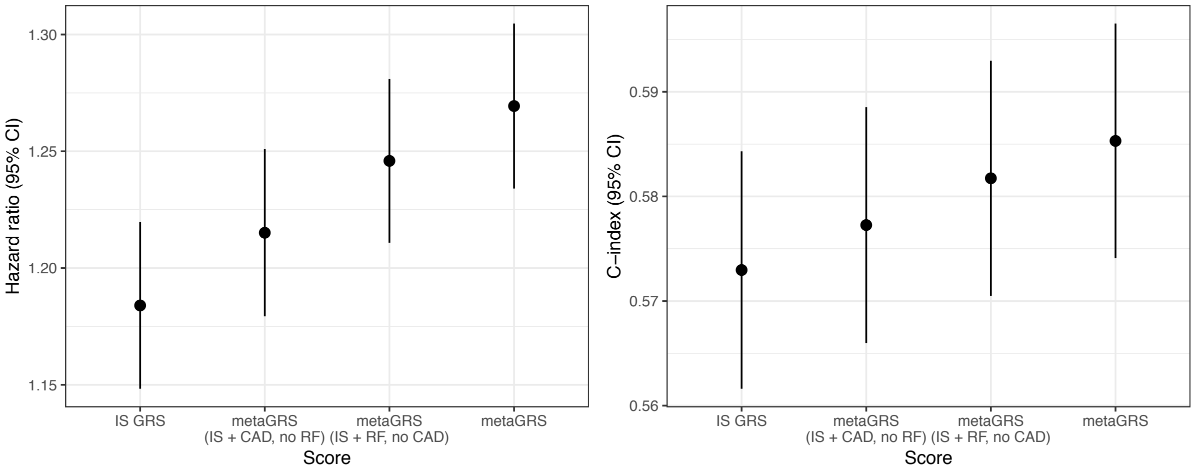

Results are for (i) the IS-only GRS; (ii) a metaGRS based on stroke-related GRSs (AS, IS, CES, LAS, SVS) and CAD-related GRSs (46K, FDR202, 1KGCAD) but no other risk factors; (iii) a metaGRS based on stroke-related GRSs (AS, IS, CES, LAS, SVS) and risk-factor-related GRSs (SBP, DBP, TC, LDL, HDL, TG, AF, BMI, Height, T2D, Smoking) but no CAD-related GRSs; (iv) the original 19-component metaGRS. Results are in the UK Biobank validation set (n=395,393). Error bars represent 95% confidence intervals.

Supplementary Figure 7: Association of established risk factors with incident ischaemic stroke, adjusted or not adjusted for the metaGRS.

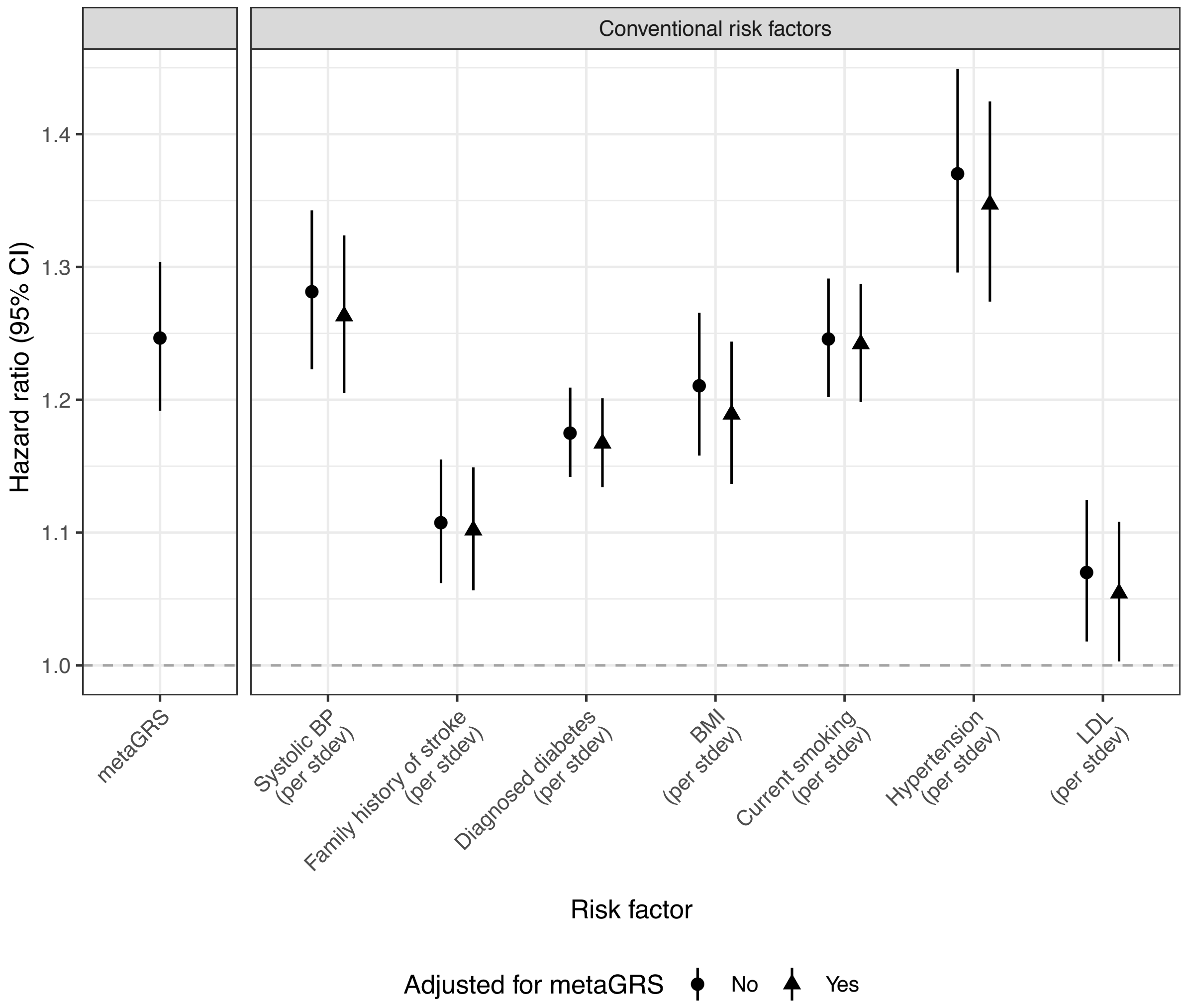

Hazard ratios (95% CI) are from Cox regression models of each risk factor (with or without the metaGRS), stratified by sex and adjusting for chip and 10 PCs. Results are presented per standard deviation of each factor, in the UKB validation dataset (n=390,849, prevalent stroke events removed). Error bars represent 95% confidence intervals.

Supplementary Figure 8: Hazard ratios of incident ischaemic stroke for the metaGRS.

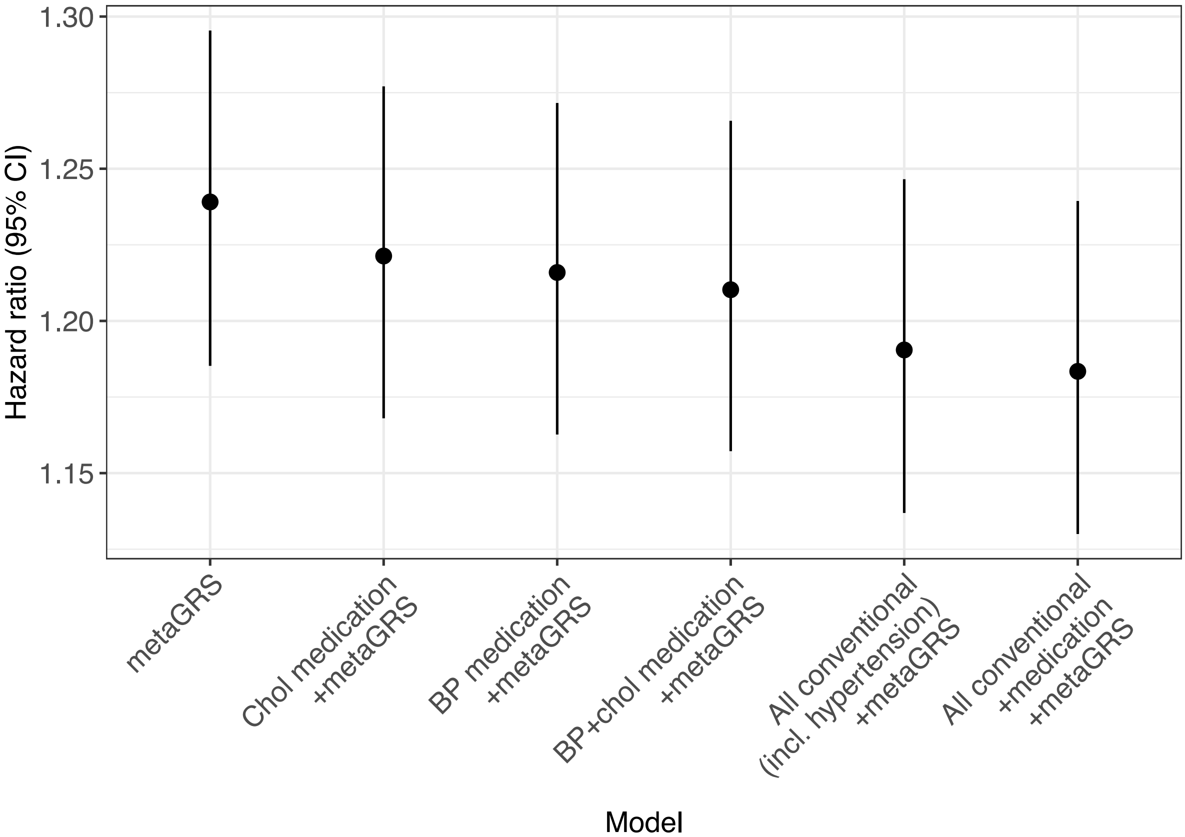

Results are for the UKB validation dataset (n=390,849, prevalent stroke events removed).

(1) '*metaGRS*': sex-stratified, adjusted for chip and 10 PCs;
(2) '*Chol medication + metaGRS*': adjusting for cholesterol-lowering medication status;
(3) '*BP medication + metaGRS*': adjusting for BP medication status;
(4) '*BP+chol medication+metaGRS*': adjusting for both cholesterol and BP lowering medications;
(5) '*All conventional+metaGRS*': adjusting for established risk factors (systolic BP, diastolic BP, family history of stroke, body mass index, current smoking, diagnosed high cholesterol, hypertension);
(6) '*All conventional+medication+metaGRS*': adjusting for all conventional risk factors as well as cholesterol-lowering and BP medication.

Error bars represent 95% confidence intervals.

Supplementary Figure 9: Calibration of logistic regression models of the metaGRS in the UK Biobank validation set (n=395,393), evaluated in deciles of predicted absolute risk of ischaemic stroke.

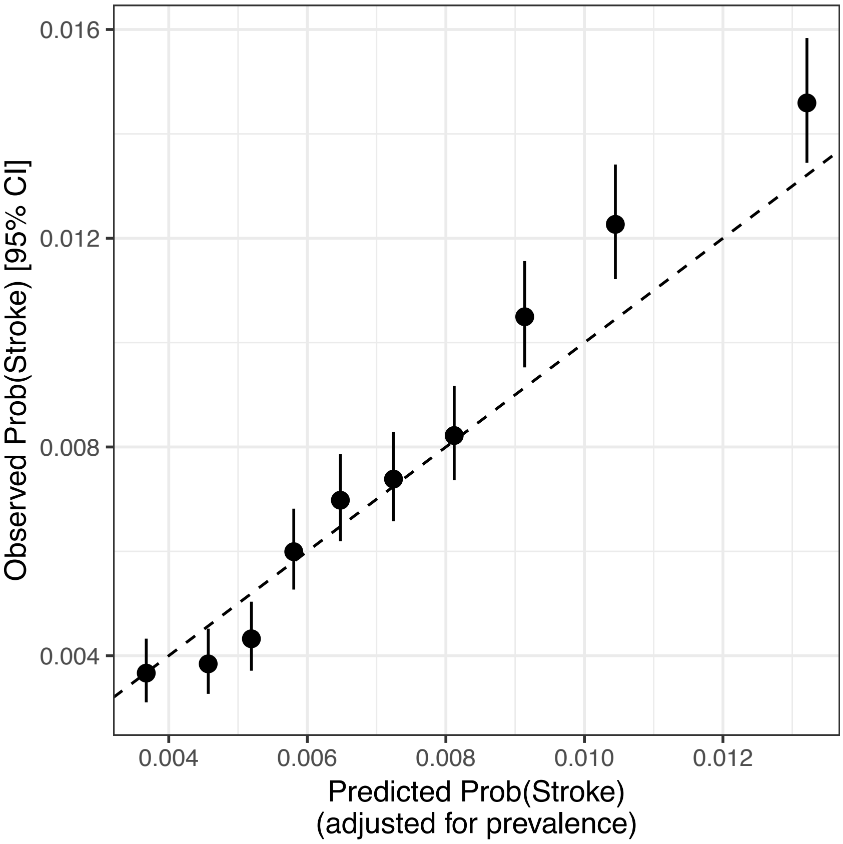

Logistic regression was performed in the n=12,000 derivation set, adjusting for chip, sex, and 10 genetic PCs. The dashed line represents perfect calibration. Error bars represent 95% confidence intervals.

Supplementary Figure 10: The heritability explained by the metaGRS, as a function of the assumed (narrow-sense) heritability of ischaemic stroke on the liability scale.

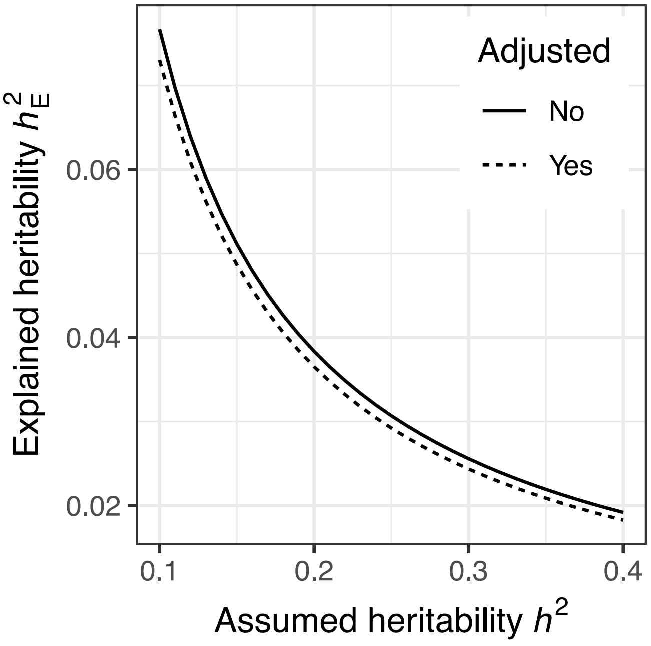

Estimates are based on linear regression, either (i) adjusted for sex, age of assessment, and 10 genetic PCs, or (ii) not adjusted for these covariates. Results are for the UKB validation set.

Supplementary Figure 11: Generalised additive model (GAM; with cubic splines) regression of the stroke metaGRS on the UK Biobank place of birth (north and east coordinates), n=381,041.

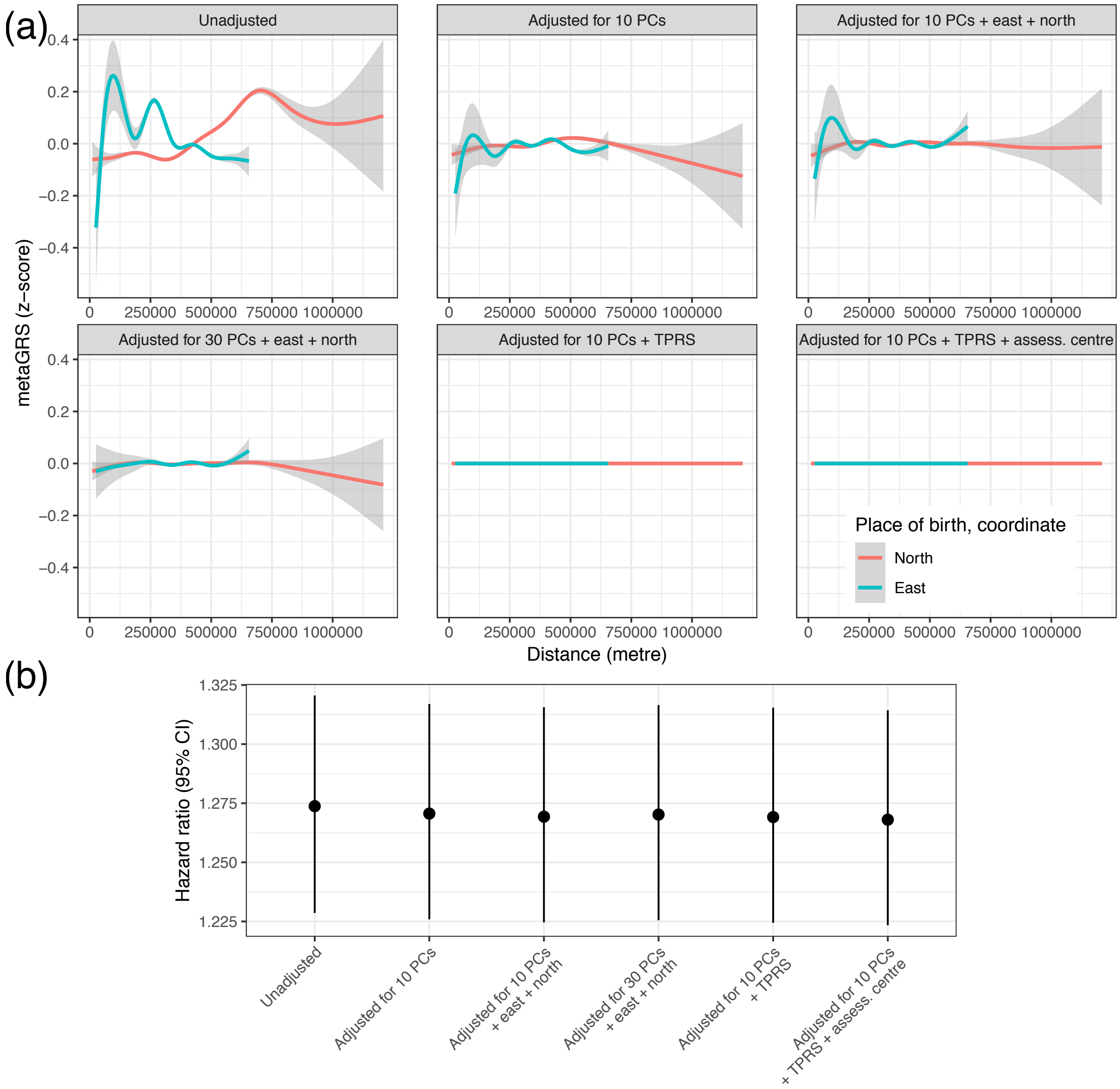

(a) Adjustments include: (i) unadjusted for geography; (ii); residuals from top 10 genetic PCs; (iii); residuals from top 10 PCs and natural splines of north and east coordinates; (iv); same as (iii) except adjusting for top 30 PCs; (v) residuals from 10 PCs and thin plate regression spline (TPRS) of the north and east coordinates; (vi) same as (v) but additionally adjusted for UKB assessment centre. Individuals without known place of birth were excluded. **(b)** Hazard ratios for the residuals of the metaGRS regressed on the above variables. Results are for the UKB validation set, excluding individuals with missing place of birth coordinates. Error bars/bands represent 95% confidence intervals.

Supplementary Tables

Supplementary Table 1: Sources of summary statistics used for the metaGRS

Category	GRS	Phenotype	Number of SNPs in score	Reference
Stroke	AS	Any stroke	3,236,236	Malik et al. [2]
	IS	Ischaemic stroke	3,284,492	Malik et al. [2]
	LAS	Large artery stroke	3,353,125	Malik et al. [2]
	SVS	Small vessel stroke	3,265,676	Malik et al. [2]
	CES	Cardioembolic stroke	3,262,206	Malik et al. [2]
Lipids	HDL	HDL cholesterol	115,051	Willer et al. [3]
	TG	Triglycerides	113,406	Willer et al. [3]
	LDL	LDL cholesterol	113,183	Willer et al. [3]
	TC	Total cholesterol	115,019	Willer et al. [3]
Cardiovascular (non-CAD)	AF	Atrial fibrillation	1,013	Weng et al. [4]
Smoking	Smoking	Cigarettes per day	895,675	Tobacco Genetics Consortium [5]
BP	SBP	Systolic BP	628,674	Wain et al. [6]
	DBP	Diastolic BP	628,601	Wain et al. [6]
Anthropometric	BMI	Body mass index	967,600	Locke et al. [7]
	Height	Height	965,305	Wood et al. [8]
Diabetes	T2D	Type 2 diabetes	4,932,042	Scott et al. [9]
CAD	46K	CAD	45,810	Abraham et al. [10]
	FDR202	CAD	199	Nikpay et al. [11]
	1KG	CAD	1,713,315	Inouye et al. [12]

Supplementary Table 2: Association of the metaGRS with established risk factors.

	Estimate	95% CI
Systolic BP (mm hg)	1.67	1.61–1.73
BMI (kg m^{-2})	0.44	0.42–0.45
LDL cholesterol (mmol L^{-1})	0.061	0.058–0.063
Family history of stroke	OR=1.06	1.05–1.07
Diagnosed diabetes	OR=1.18	1.16–1.19
Current smoking	OR=1.06	1.05–1.07
Diagnosed hypertension	OR=1.20	1.19–1.21

For continuous outcomes (SBP, BMI), the associations are from linear regression; for discrete outcomes the associations are from logistic regression. All regression models were adjusted for sex, chip, age at assessment, and 10 genetic PCs, using the validation subset of UKB (incident cases and non-cases, n=390,849).

References

1. Rutten-Jacobs L*, et al.* Genetic risk, incident stroke, and the benefits of adhering to a healthy lifestyle: follow-up of 306,473 UK Biobank participants. *BMJ* **363**, k4168 (2018).
2. Malik R*, et al.* Multiancestry genome-wide association study of 520,000 subjects identifies 32 loci associated with stroke and stroke subtypes. *Nat Genet* **50**, 524-537 (2018).
3. Willer CJ*, et al.* Discovery and refinement of loci associated with lipid levels. *Nat Genet* **45**, 1274-1283 (2013).
4. Weng LC*, et al.* Genetic Predisposition, Clinical Risk Factor Burden, and Lifetime Risk of Atrial Fibrillation. *Circulation* **137**, 1027-1038 (2018).
5. Tobacco Genetics Consortium. Genome-wide meta-analyses identify multiple loci associated with smoking behavior. *Nature Genetics* **42**, 441-447 (2010).
6. Wain LV*, et al.* Novel Blood Pressure Locus and Gene Discovery Using Genome-Wide Association Study and Expression Data Sets From Blood and the Kidney. *Hypertension*, (2017).
7. Locke AE*, et al.* Genetic studies of body mass index yield new insights for obesity biology. *Nature* **518**, 197-206 (2015).
8. Wood AR*, et al.* Defining the role of common variation in the genomic and biological architecture of adult human height. *Nat Genet* **46**, 1173-1186 (2014).
9. Scott RA*, et al.* An Expanded Genome-Wide Association Study of Type 2 Diabetes in Europeans. *Diabetes* **66**, 2888-2902 (2017).
10. Abraham G*, et al.* Genomic prediction of coronary heart disease. *Eur Heart J* **37**, 3267-3278 (2016).
11. Nikpay M*, et al.* A comprehensive 1,000 Genomes-based genome-wide association meta-analysis of coronary artery disease. *Nat Genet* **47**, 1121-1130 (2015).
12. Inouye M*, et al.* Genomic Risk Prediction of Coronary Artery Disease in 480,000 Adults: Implications for Primary Prevention. *J Am Coll Cardiol* **72**, 1883-1893 (2018).
