# Supplementary material for: Genomic risk score offers predictive performance comparable to clinical risk factors for ischaemic stroke
Source: Nat Commun. 2019 Dec 20;10:5819. doi: 10.1038/s41467-019-13848-1 (PMC6925280; doi:10.1038/s41467-019-13848-1)
Supplement: Supplementary file 3 — Reporting Summary [file 41467_2019_13848_MOESM3_ESM.pdf]

nature research

Corresponding author(s): Gad Abraham, Michael Inouye, Martin Dichgans

Last updated by author(s): 24/11/19

Reporting Summary

Nature Research wishes to improve the reproducibility of the work that we publish. This form provides structure for consistency and transparency in reporting. For further information on Nature Research policies, see Authors & Referees and the Editorial Policy Checklist.

Statistics

For all statistical analyses, confirm that the following items are present in the figure legend, table legend, main text, or Methods section.

n/a	Confirmed	
☐	☒	The exact sample size (*n*) for each experimental group/condition, given as a discrete number and unit of measurement
☐	☒	A statement on whether measurements were taken from distinct samples or whether the same sample was measured repeatedly
☐	☒	The statistical test(s) used AND whether they are one- or two-sided<br>*Only common tests should be described solely by name; describe more complex techniques in the Methods section.*
☐	☒	A description of all covariates tested
☐	☒	A description of any assumptions or corrections, such as tests of normality and adjustment for multiple comparisons
☐	☒	A full description of the statistical parameters including central tendency (e.g. means) or other basic estimates (e.g. regression coefficient) AND variation (e.g. standard deviation) or associated estimates of uncertainty (e.g. confidence intervals)
☐	☒	For null hypothesis testing, the test statistic (e.g. *F*, *t*, *r*) with confidence intervals, effect sizes, degrees of freedom and *P* value noted<br>*Give P values as exact values whenever suitable.*
☒	☐	For Bayesian analysis, information on the choice of priors and Markov chain Monte Carlo settings
☒	☐	For hierarchical and complex designs, identification of the appropriate level for tests and full reporting of outcomes
☐	☒	Estimates of effect sizes (e.g. Cohen's *d*, Pearson's *r*), indicating how they were calculated

Our web collection on statistics for biologists contains articles on many of the points above.

Software and code

Policy information about availability of computer code

Data collection	No new data was collected. We used PLINK 1.9 (v2018-02-21) to perform genetic quality control, LD thinning, and to generate polygenic risk scores. We used KING v2.1.4 to perform genetic relatedness analysis.
Data analysis	Data was analysed using R v3.2.2, including the packages glmnet (v2.0-16) and survival (v2.43-1).

For manuscripts utilizing custom algorithms or software that are central to the research but not yet described in published literature, software must be made available to editors/reviewers. We strongly encourage code deposition in a community repository (e.g. GitHub). See the Nature Research guidelines for submitting code & software for further information.

Data

Policy information about availability of data

All manuscripts must include a data availability statement. This statement should provide the following information, where applicable:
- Accession codes, unique identifiers, or web links for publicly available datasets
- A list of figures that have associated raw data
- A description of any restrictions on data availability

This study used data from the publicly available UK Biobank project. The stroke metaGRS is freely available at https://dx.doi.org/10.6084/m9.figshare.8202233.

Field-specific reporting

Please select the one below that is the best fit for your research. If you are not sure, read the appropriate sections before making your selection.

☒ Life sciences ☐ Behavioural & social sciences ☐ Ecological, evolutionary & environmental sciences

For a reference copy of the document with all sections, see nature.com/documents/nr-reporting-summary-flat.pdf

Life sciences study design

All studies must disclose on these points even when the disclosure is negative.

Sample size	No power calculations were performed, as we used all the relevant UK Biobank data which was available. The final sample size was n=11,995 individuals for the derivation set and n=395,393 individuals for the validation set.
Data exclusions	We excluded participants who were not of British white ancestry (according to the principal component analysis), participants without matching genotypes, participants who had withdrawn their consent, and participants with self-reported stroke at age <20 years due to the potential unreliability of such records. We excluded genetic markers from the UK10K/1000Genomes imputation reference panel due to known issues with these markers, as well as single nucleotide polymorphisms with MAF<0.1%.
Replication	The polygenic risk scores were based on studies that did not include the UK Biobank. The polygenic risk scores were tuned on a derivation subset of the UK Biobank. The final stroke metaGRS was evaluated on the validation set, which did not include the derivation set.
Randomization	There was no randomisation as the UK Biobank is an observational study. The analyses controlled for covariables including sex, 10 genetic principal components, and genotyping chip. The survival analysis also adjusted for age using an age-as-time-scale approach.
Blinding	Blinding is not relevant to this study as no interventions were performed.

Reporting for specific materials, systems and methods

We require information from authors about some types of materials, experimental systems and methods used in many studies. Here, indicate whether each material, system or method listed is relevant to your study. If you are not sure if a list item applies to your research, read the appropriate section before selecting a response.

Materials & experimental systems

n/a	Involved in the study	
☒	☐	Antibodies
☒	☐	Eukaryotic cell lines
☒	☐	Palaeontology
☒	☐	Animals and other organisms
☐	☒	Human research participants
☒	☐	Clinical data

Methods

n/a	Involved in the study	
☒	☐	ChIP-seq
☒	☐	Flow cytometry
☒	☐	MRI-based neuroimaging

Human research participants

Policy information about studies involving human research participants

Population characteristics	We used the UK Biobank phase 2 genotype data, together with linked phenotype information (either self reported, collected at UK Biobank assessment, or from hospital records), out of which we selected the British white individuals from principal component analysis (n=407,388 after sample exclusions).
Recruitment	Participants were recruited by the UK Biobank project.
Ethics oversight	Analyses were approved by the UK Biobank under project 26865.

Note that full information on the approval of the study protocol must also be provided in the manuscript.
